# Urinary neutrophil gelatinase‐associated lipocalin determines short‐term mortality and type of acute kidney injury in cirrhosis

**DOI:** 10.1002/jgh3.12377

**Published:** 2020-07-03

**Authors:** Suhas Udgirkar, Pravin Rathi, Nikhil Sonthalia, Sanjay Chandnani, Qais Contractor, Ravi Thanage, Samit Jain

**Affiliations:** ^1^ Department of Gastroenterology & Hepatology Topiwala National Medical College and B.Y.L Nair hospital Mumbai Mumbai Maharashtra India; ^2^ Department of Gastroenterology and Hepatology Bombay Hospital Institute of Medical Sciences (BHIMS) & Topiwala National Medical College and B.Y.L Nair hospital Mumbai Maharashtra India

**Keywords:** acute kidney injury, biomarker, hepatorenal syndrome, spontaneous bacterial peritonitis

## Abstract

**Background and Aim:**

Acute kidney injury increases mortality in cirrhotic patients by four fold. This study aimed to determine the usefulness of urinary neutrophil gelatinase‐associated lipocalin (uNGAL) for differential diagnosis for acute kidney injury and for predicting short‐term mortality in cirrhotic patients.

**Methods:**

We enrolled 94 patients of decompensated cirrhosis. uNGAL was measured upon hospital admission in all patients. Patients with urinary tract infection and anuria were excluded. Patients were followed for 30 days or until death.

**Results:**

Ten (9%) patients had normal kidney function, 9 (11.37%) stable chronic kidney disease, 32 (29.50%) prerenal azotemia, 33 (36.37%) hepatorenal syndrome (HRS), and 10 (13.64%) intrinsic acute kidney injury (iAKI). Prerenal azotemia had lower median uNGAL values compared to HRS and iAKI (95.50 *vs* 465.00 *vs* 1217.50, *P* < 0.001). uNGAL levels were significantly higher in patients who died within 30 days (717.17 ± 494.26 *vs* 331.65 ± 313.65 ng/mL, *P* −0.0017). On univariate analysis, serum creatinine (sCr), uNGAL, Model for End‐Stage Liver Disease (MELD) score on admission, and length of stay were significant, and on multivariate analysis, uNGAL and hepatic encephalopathy (HE) were significant in predicting mortality.

**Conclusions:**

uNGAL at baseline serves as an early marker in differentiating HRS, prerenal AKI, and iAKI in cirrhotic patients, where sCr values are not useful. Patients with higher uNGAL levels had higher transplant‐free mortality at 30 days.

## Graphical abstract

uNGAL at baseline serves as early marker in differentiating HRS, prerenal AKI and intrinsic AKI in cirrhotics, where sr. creatinine values are not useful. Patients with higher uNGAL levels had higher transplant free mortality at 30 days

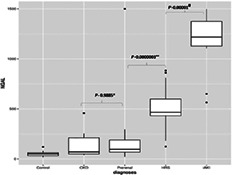

## Introduction

Acute kidney injury (AKI) is a known lethal complication of liver cirrhosis. Up to 20% of hospitalized patients with cirrhosis develop AKI.^1^, ^2^ Types of AKI in cirrhosis include prerenal azotemia, hepatorenal syndrome (HRS), and intrinsic AKI (iAKI). Clinical presentations and mortality risks for each of these varies significantly. The interpretation of serum creatinine (sCr) level in cirrhosis can be confounded by malnutrition and reduced muscle mass often observed in patients with severe liver disease. Other potential pitfalls may include abnormal body distribution of creatinine in subjects with ascites and significant peripheral edema, as well as interference of creatinine assays due to hyperbilirubinemia. These limitations of sCr highlight the need for improved diagnostic methods to determine the type of AKI in cirrhosis.^3^, ^4^, ^5^

While acute tubular necrosis (ATN) is characterized by alterations in renal tubular cells, HRS is due to the functional impairment of kidney function related to intense vasoconstriction of the kidney circulation in the absence of significant histological lesions.^6^, ^7^, ^8^, ^9^, ^10^, ^11^ Accurate diagnosis of the causes of acute impairment of kidney function in cirrhosis is important as treatment and outcome vary significantly. In view of the lack of an ideal biomarker for kidney dysfunction that rapidly and accurately discriminates HRS from other forms of AKI, the AKI International Ascites Club (AKI IAC) has proposed the 48‐h diagnostic algorithm.^12^ Prerenal azotemia should be treated with plasma volume expansion, but this is not effective and may be even deleterious in patients with ATN.^12^, ^13^, ^14^, ^15^ Hence, there is an urgent need for an ideal biomarker to improve the diagnostic accuracy of AKI in cirrhosis.

Neutrophil gelatinase‐associated lipocalin (NGAL) is a 25‐kD protein expressed by injured kidney tubular epithelia.^16^ Plasma and urine NGAL (uNGAL) have been shown to be useful markers in a number of clinical settings in terms of prediction of development of AKI and mortality.^17^, ^18^, ^19^ However, data are scarce regarding the role of uNGAL in cirrhosis and HRS.^20^, ^21^, ^22^ Levels of uNGAL rise exponentially early in the course of AKI before sCr elevation.^23^, ^24^ Volume status, diuretic use, or prerenal azotemia does not alter uNGAL levels. In addition, nonprogressive chronic kidney disease (CKD) does not induce uNGAL expression.^25^Finally, growing evidence also suggests that elevated uNGAL levels independently predict clinical outcomes, including short‐term mortality.^19^, ^25^, ^26^

Previous studies in patients of HRS have determined various prognostic markers as predictors of short‐ and long‐term mortality, which included sCr, low urine sodium, advanced age, MELD score, Cystatin C, etc.^27^, ^28^, ^29^ Hence, we undertook this study to determine the usefulness of uNGAL in differentiating different types of AKI in cirrhosis and also to identify its role as a prognostic marker for predicting short‐term (30‐day) transplant‐free mortality.

## Materials and Methods

### *Study protocol*

This was a prospective observational study conducted over a 1‐year period (January 2016 to January 2017). Hospitalized patients were included and followed for at least 3 month post admission or until death, whichever is earlier. The study protocol was approved by the Institutional Ethics Committee.

Patients were diagnosed as having cirrhosis on the basis of established clinical, laboratory, and imaging criteria with or without liver biopsy. Sample size was calculated using the Buderer formula. Considering an expected sensitivity of 75% and precision of 10%, the sample size was calculated (AKI, including prerenal, HRS, iAKI) to be 75; in addition, 10 CKD patients and 10 patients with chronic liver disease (CLD) without AKI for comparison were enrolled, and hence, the total expected sample size was 95. We enrolled 75 patients with renal injury, and 9 patients with CKD, and 10 were controls (CLD without AKI), hence, the total sample size was 94 patients. We screened 129 consecutive patients and included 94 of these patients. Patients on chronic hemodialysis (HD), those who had urinary tract infection (UTI) (urine WBC > 10 per high power field or positive urine culture) or urinary obstruction, and patients unwilling to participate were excluded. Patients were followed from admission till day 30 or death, whichever was earlier. Demographic, clinical, and laboratory data were collected at the time of admission. Urine for uNGAL measurement was collected once at the time of admission for all patients. sCr was prospectively measured, and adjudication of kidney function category was performed by a hepatologist and nephrologist. For patients with impaired kidney function (Cr > 1.5 mg/dL or increase in creatinine by 0.3 mg in 48 hours), diuretics were halted at least for 48 hours, and patients were infused with albumin and/or saline when appropriate to exclude prerenal AKI as a result of volume depletion.^3^, ^19^ To differentiate acute tubular necrosis (ATN)/intrinsic AKI (iAKI) from HRS, those with 1) presence of tubular epithelial cells on urinalysis, 2) urine sodium >10 mEq/L, and 3) those that did not meet the ICA‐HRS criteria were considered to have iAKI.^3^ Renal and collecting system ultrasound was performed to exclude a postrenal cause for renal dysfunction. Patients with CLD with normal kidney function (n=10) were enrolled in this study as controls to compare levels of uNGAL in this population (Fig. S1‐flow diagram).

### *Definitions of kidney disease*

#### *Normal kidney function*

Stable sCr < 1.5 mg/dL and < 0.3 mg/dL above baseline.

#### *Definition of AKI*

Increase in sCr ≥0.3 mg/dL (≥26.5 mmol/L) within 48 hours or a percentage increase in sCr ≥50% from baseline, which is known, or presumed, to have occurred within the previous 7 days.

#### *Prerenal azotemia*

A transient increase in sCr to >1.5mg/dL and 0.3 mg/dL above baseline, with subsequent decrease in sCr to <1.5 mg/dL or to mean baseline creatinine within 48 hours of treatment with diuretic withdrawal and intravenous hydration.

#### *HRS Defined by the international club of ascites (ICA‐AKI) guidelines*

AKI as defined above and including the presence of cirrhosis and ascites that failed to improve after 48 hours of diuretic withdrawal and volume expansion with albumin in the absence of shock, nephrotoxic medications, or parenchymal kidney disease, as suggested by proteinuria (500 mg/day, microhematuria [>50 red blood cells/high powered field] and/or abnormal kidney imaging).

#### *Stable chronic kidney disease*

Estimated glomerular filtration rate (eGFR) <60 mL/min over 3 months prior to hospital admission and serum creatinine 0.3 mg/dL above baseline.

#### *Intrinsic (ATN)*

Defined as acute elevation in sCr to >1.5 mg/dL and 0.3 mg/dL above baseline, not responding within 48 hours to volume resuscitation and not meeting the criteria for HRS (Fig. S1).^12,22^

### *Determination of* *uNGAL* *levels*

Early‐morning fresh urine samples were collected. These samples were stored at 2–8°C for 24 hours. Samples were centrifuged at 400 rpm for 5 min, and aliquots were stored. uNGAL values were determined by the ARCHITECT chemiluminescent microparticle immunoassay (CMIA) method.^30^ In the first step, sample and wash buffer were combined to create a 1:10 sample dilution. An aliquot of the prediluted sample, wash buffer, and anti‐NGAL‐coated paramagnetic microparticles were combined. NGAL present in the sample binds to anti‐NGAL‐coated microparticles, and the reaction mixture was washed. In the second step, anti‐NGAL acridinium‐labeled conjugate was added. Following another wash cycle, pretrigger and trigger solutions were added to the reaction mixture. The resulting chemiluminescent reaction was measured in relative light units (RLUs). A relationship exists between the amount of NGAL in the sample and the RLUs detected by the ARCHITECT *I* System optics. sCr was assayed in the clinical laboratory by the Jaffe reaction. Urine creatinine was measured by colorimetric assay and was used to calculate eGFR with the Modification of Diet in Renal Disease (MDRD) formula.^31^ Urine sodium was measured by ion‐selective electrode assay and used to determine fractional excretion of sodium (FeNa).

### *Statistical analysis*

All data were analyzed using SPSS statistics version 10 (SPSS Inc., Chicago, IL, USA). The student's *t*‐test was used for the analysis of the binary group of normally distributed variables. The Mann–Whitney U‐test was used for nonparametric binary assessments. One‐way ANOVA test for multigroup variance analysis was used. Values were given as median ± IQ/mean ± SD. As biomarker levels were not clearly normally distributed, receiver operating characteristic (ROC) curves were generated to determine biomarker (uNGAL, sCr, and FeNa) cut‐offs, which maximized their sensitivity and specificity in the differentiation of the type of AKI. All variables that predicted mortality with *P* < 0.2 or were thought to be central to the analysis were included in the multivariable model building. Variables that were no longer significant at *P* > 0.05 or not essential to the analysis were sequentially removed. As MELD was calculated directly from sCr values, these two variables were not included in the same multivariable models (R^2^ = 0.5). An ROC curve analysis was performed to identify the sensitivity and specificity of cut‐off values of biomarkers.

## Results

### *Characteristics of the patient population*

Demographic and clinical data, detailed characteristics of liver function tests, kidney function, urine parameters, and outcome of patients were divided into four categories according to the cause of impairment in kidney function, shown in Table 1.

**Table 1:** Depicting demographic, clinical, and biochemical parameters and outcome of patients at day 30

Variable	Total	Control (10)	Prerenal azotemia (32)	Hepatorenal syndrome (33)	Intrinsic AKI (10)	Chronic kidney disease (9)	*P*‐value
Age (mean [SD])	47.3 (12.03)	50.50 (10.23)	46.94 (10.60)	47.88 (13.75)	42.20 (14.47)	42.44 (9.38)	0.619
Gender, m (%)	67	4 (40.0)	22 (68.7)	27 (81.8)	7 (70.0)	4 (44.4)	0.280
Cause of Cirrhosis Hepatitis B/HCV/ALD/AIH/Idiopathic	18/22/38/10/2	3/3/3/1/0	5/6/13/4/4	7/8/13/3/2	0/3/6/1/0	3/2/3/1/0	0.082
Ascites (%)	89	9 (90.0)	30 (93.8)	33 (100.0)	10 (100.0)	7 (77.8)	0.258
SBP (%)	13	1 (10.0)	4 (12.5)	6 (18.2)	2 (20.0)	0 (0.0)	0.652
Hepatic encephalopathy (%)	23	2 (20.0)	6 (18.8)	8 (24.2)	5 (50.0)	1 (11.1)	0.270
HCC (%)	5	0 (0.0)	0 (0.0)	2 (6.0)	1 (10.0)	2 (22.2)	0.009
TLC (per liter) (mean [SD])	9064.90 (5939.8)	6870.00 (2652.48)	10 021.88 (7191.65)	8242.87 (5554.38)	11 340.00 (6887.70)	6944.44 (2674.47)	0.260
T.Bil (mg/dL) (mean [SD])	5.67 (6.25)	4.48 (4.41)	5.45 (6.05)	6.21 (7.31)	7.73 (6.60)	3.49 (3.77)	0.596
Albumin (g/dL) (mean [SD])	2.67 (0.52)	2.65 (0.39)	2.54 (0.44)	2.65 (0.65)	2.51 (0.37)	2.82 (0.50)	0.607
INR (mean [SD])	1.31 (0.4)	1.09 (0.14)	1.27 (0.30)	1.41 (0.51)	1.45 (0.43)	1.13 (0.15)	0.078
MELD (mean [SD])	20.3 (7.28)	13.10 (4.09)	18.62 (6.72)	23.30 (6.79)	26.60 (6.17)	16.22 (3.53)	<0.001
CTP (mean [SD])	9.1 (1.17)	8.40 (1.17)	9.06 (1.11)	9.42 (1.03)	9.90 (1.10)	8.22 (1.09)	0.002
Length of stay (days) (mean [SD])	9.2 (2.3)	6.10 (3.18)	7.81 (4.28)	14.61 (4.68)	12.20 (7.10)	10.44 (8.96)	<0.001
sCr (mg/dL) (mean [SD])	1.54 (1.12)	1.00 (0.27)	1.63 (0.77)	2.58 (0.93)	3.32 (1.91)	1.84 (0.71)	<0.001
GFR (mean [SD])	41.55 (21.22)	65.15 (20.04)	49.35 (22.86)	31.72 (17.36)	24.01 (15.07)	43.14 (24.53)	<0.001
Serum Na(mEq/L) (mean [SD])	131.73 (98.15)	133.40 (6.95)	132.09 (8.04)	131.24 (8.35)	129.80 (9.51)	132.56 (8.78)	0.875
Urine Na(mEq/L) (mean [SD])	1.21 (0.82)	38.00 (13.56)	42.71 (19.58)	64.24 (22.35)	99.00 (21.15)	55.70 (21.83)	<0.001
Urine Cr (mg/dL) (mean [SD])	76.72 (31.60)	54.70 (18.17)	65.89 (26.62)	92.82 (28.49)	99.20 (38.61)	55.66 (16.79)	<0.001
uNGAL (ng/mL) (median [IQ])	375.65 (71.50,544.00)	50.30 (32.25, 62.75)	95.50 (67.75, 189.68)	465.00 (430.00, 599.00)	1217.50 (1128.00, 1375.00)	70.00 (48.00, 210.00)	<0.001
FeNa (mean [SD])	1.21 (0.82)	0.60 (0.14)	0.81 (0.35)	0.85 (0.66)	2.93 (0.80)	1.15 (0.24)	<0.001
Outcome 30 (d [%])	18	0 (0.0)	2 (6.2)	13 (39.36)	3 (36.33)	(0.0)	0.006

AIH, autoimmune hepatitis; AKI, acute kidney injury; ALD, alcoholic liver disease; CTP, Child–Pugh score; FeNa, fractional excretion of sodium; GFR, glomerular filtration rate; HCC, hepatocellular carcinoma; HCV, hepatitis C; INR, international normalized ratio; MELD, Model for End‐Stage Liver Disease; SBP, spontaneous bacterial peritonitis; sCr, serum creatinine; T.Bil, total bilirubin; TLC, total leukocyte count; U.Cr, urine creatinine; Urine Na, urine sodium; uNGAL, urinary neutrophil gelatinase‐associated lipocalin.

After adjudication of sCr levels in 84 patients with AKI, 33 had HRS and prerenal in 32, 10 had iAKI, and 9 had stable CKD (KDIGO class 3 in 5 and class 2 in 4 patients). Mean age in all four groups was 46.8 years.

The most common cause of cirrhosis was alcohol (40.5%) followed by hepatitis C (23.4%), hepatitis B (19.15%), autoimmune hepatitis (10.65%), and other causes (7.45%). Thirteen patients had spontaneous bacterial peritonitis, and 22 had hepatic encephalopathy. Five patients had underlying hepatocellular carcinoma (HCC).

On post hoc analysis, mean Model for End‐Stage Liver Disease (MELD) levels were higher in the iAKI and HRS groups than the prerenal and CKD groups, and the difference was statistically significant (*P* < 0.001) (Table S1).

sCr levels calculated on admission when AKI was already present or after development of AKI showed similar values in the HRS and iAKI groups. Hence, sCr could not differentiate HRS from iAKI.

**Figure 1:**
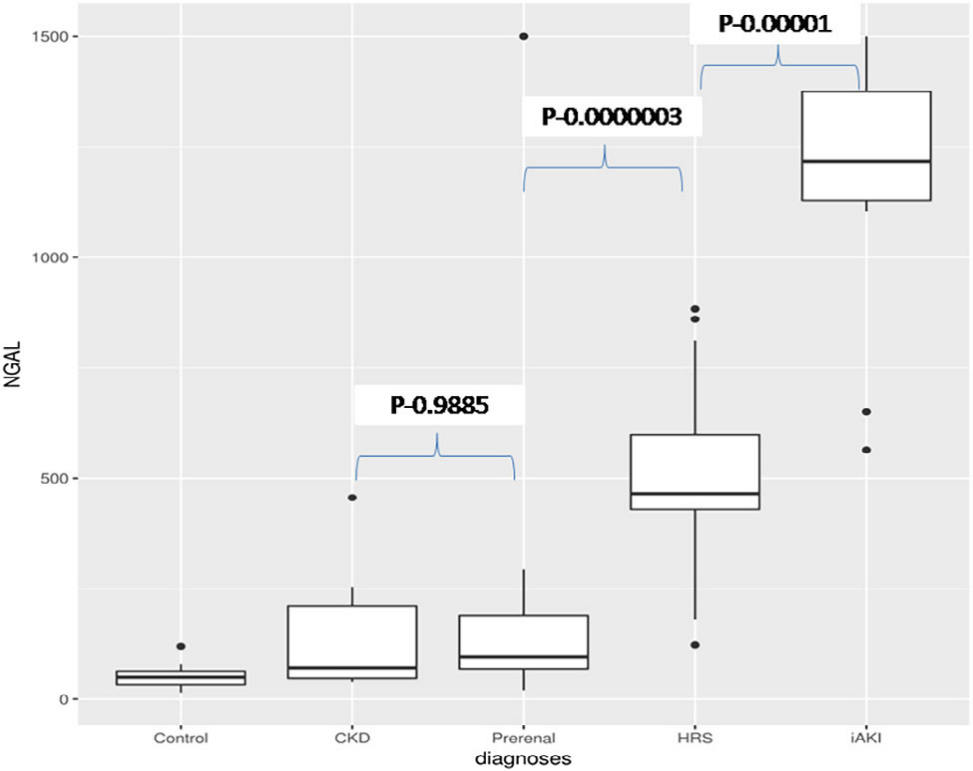
Box plot showing uNGAL values in different types of acute kidney injury.

Median uNGAL levels in the HRS group of 465.00 (430.00, 599.0) ng/ml were intermediate between iAKI, 1217.50 (1128.00, 1375.00) ng/ml, where they were highest, and prerenal failure, 95.50 (67.75, 189.68) ng/ml, where they were lowest (Table 1) (Fig. 1).

Mean values of uNGAL in patients with and without spontaneous bacterial peritonitis (SBP) were not statistically different, 608 ng/mL and 354.09 ng/mL, respectively (*P* = 0.103). Five patients had pneumonia, and three had cellulitis (two also had SBP), and uNGAL levels in each of these groups were also similar. Similar results were seen in patients with or without hepatic encephalopathy (*P* = 0.078). Baseline sCr was higher in the iAKI and HRS groups but not significantly different (2.52 mg/dL *vs* 3.32 mg/dL). Hence, early identification of the type of AKI was not possible based on sCr level. For this purpose, uNGAL levels were calculated at the time of development of AKI, and values were compared between all four groups.

## Diagnostic Accuracy of uNGAL and sCr

### *ATN (iAKI)* versus *HRS*

**Figure 2:**
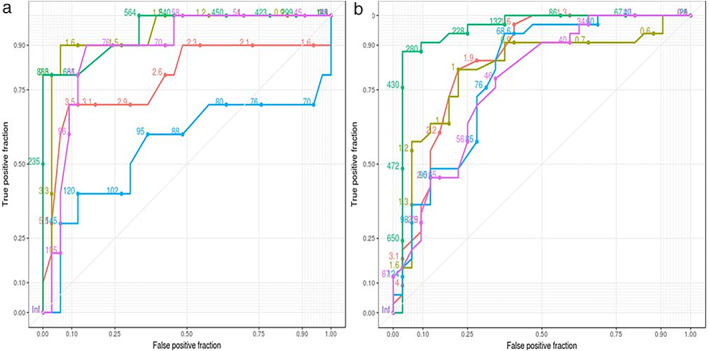
(a) Graph with receiver operating characteristics (ROC) curves for comparison of parameters between HRS and ATN (intrinsic AKI). (b) Graph showing ROC curves for comparison of parameters between HRS and prerenal AKI. (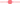) Creat, (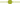) FeNa, (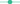) NGAL, (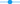) urine_creat, and (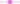) urine_na.

The AUROC for uNGAL was 0.94, and the optimal cut‐off point was determined to be 650 ng/mL (sensitivity of 100% and specificity of 83.78%, positive likelihood ratio–6.5, and negative likelihood ratio–0). Thus, if the uNGAL is less than 650 ng/mL, a diagnosis of HRS is likely, and the diagnosis will be ATN (iAKI) when values are greater than 650 ng/mL (Fig. 2a and Table 2).

**Table 2:** Sensitivity, specificity, positive likelihood ratio (PLR) and negative likelihood ratio (NLR) of parameters in differentiating hepatorenal syndrome (HRS) from intrinsic acute kidney injury (iAKI) and HRS from prerenal AKI

Biomarker (cutoff)	AUROC	Sensitivity (%)	Specificity (%)	PLR (%)	NLR (%)
HRS *vs* iAKI					
uNGAL (≥650 ng/mL)	0.95	100	83.78	6.5	0
sCr (≥4 mg/dL)	0.78	70	90	7.0	3.3
Prerenal *vs* HRS					
uNGAL (≥299 ng/mL)	0.94	87.90	96.30	3.73	0.16
sCr (≥2 mg/dL)	0.78	81.0	78.00	2.2	0.29

sCr, serum creatinine; uNGAL, urinary neutrophil gelatinase‐associated lipocalin.

sCr had the lower sensitivity and specificity to differentiate iAKI from HRS. (Fig. 2a and Table 2).

### *Prerenal* versus *HRS*

uNGAL values of more than 299 ng/mL were able to differentiate prerenal and HRS with sensitivity and specificity of 87.9% and 96.3%, respectively, and sCr had a sensitivity and specificity of 81% and 78%, respectively (Fig. 2b and Table 2).

### *uNGAL* *in predicting mortality*

**Figure 3:**
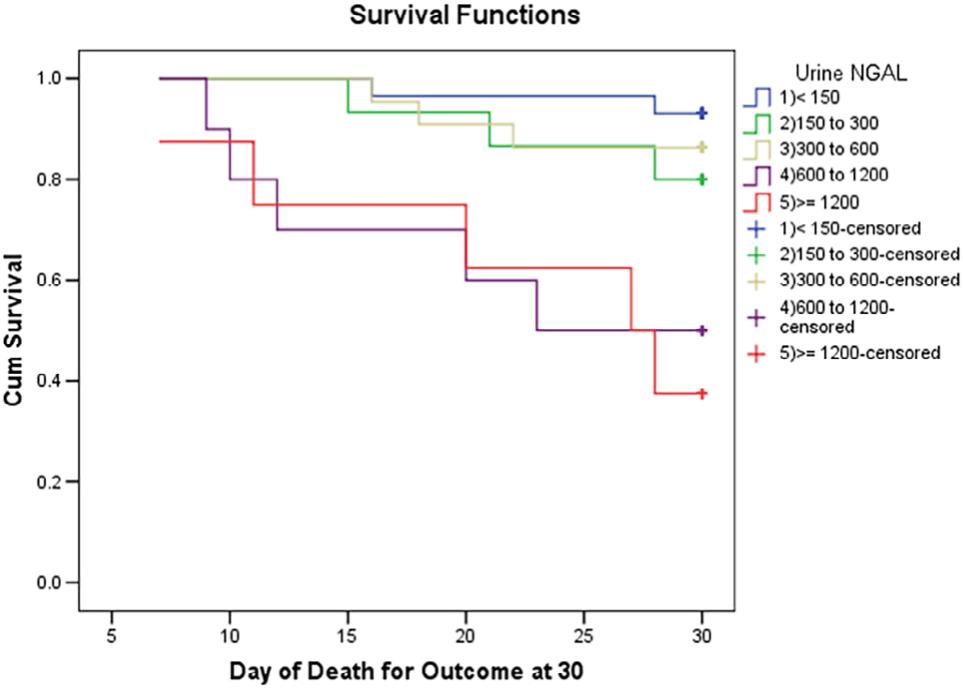
Survival analysis of patients at 30 days according to urinary NGAL levels. (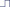) <150, (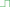) 150 to 300, (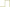) 300 to 600, (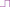) 600 to 1200, (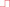) ≥1200, (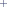) <150‐censored, (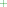) 150 to 300‐censored, (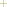) 300 to 600 censored, (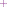) 600 to 1200‐censored, and (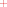) ≥1200‐censored.

Eighteen patients died at follow up of 30 days and 22 patients at 90 days. Mortality at 30 days in the HRS group was 39.30% (13 cases) and in the iAKI group was 36.36% (3 cases), with zero and two patients in the CKD and prerenal groups, respectively. Patients who died at 30 days had higher uNGAL levels than those who were alive (*P* = 0.00338). On univariate analysis, uNGAL, sCr after 48 h, MELD level at baseline, and length of stay in hospital were statistically significant in predicting mortality at 30 days (Table 3). As MELD levels include sCr, it was not measured separately in multivariate analysis (Table 4). Although HCC was a confounder and can cause increased mortality, only five patients had HCC, and 30‐day mortality may not be affected by HCC; thus, it was not excluded from analysis. On multivariate analysis, only uNGAL and hepatic encephalopathy were found to be statistically significant. On survival analysis, values of NGAL correlated with survival, patients were divided into five groups according to uNGAL levels, and increasing uNGAL level had decreased survival at 30 days (Fig. 3).

**Table 3:** Comparison of parameters in survivors and nonsurvivors (univariate analysis)

	Survivors (66)	Nonsurvivors (18)	*P* value
S. Albumin (g/L)	2.64 ± 0.08	2.48 ± 0.38	0.190
uNGAL(ng/mL)	331.65 ± 313.65	717.17 ± 494.26	0.0017
sCr (mg/dL)	1.98 ± 1.28	3.18 + 1.08	0.0002
CTP	9.04 ± 1.07	9.50 ± 1.47	0.222
MELD	19.16 ± 3.1	25.33 ± 2.7	0.004
SBP (no.)	8 (33.33%)	4 (66.66)	0.276
Hepatic encephalopathy(no.)	9 (55.00%)	11 (45.00%)	0.278
Length of stay in hospital (days)	9.5 days	15.5 days	0.002

CTP, Child–Pugh score; MELD, model for end‐stage liver disease; sCr, serum creatinine; uNGAL, urinary neutrophil gelatinase‐associated lipocalcin.

**Table 4:** Logistic regression with outcome at day 30 as dependent variable

Variables	B	SE	Wald	*P*‐value	95.0% CI for EXP(B)	
					Lower	Upper
MELD	0.098	0.058	2.816	0.093	0.984	1.236
CTP	−0.317	0.365	0.758	0.384	0.356	1.487
Urine NGAL	0.002	0.001	7.082	0.0078	1.001	1.004
Spontaneous Bacterial Peritonitis (Yes)	−0.143	0.939	0.023	0.879	0.138	5.454
Hepatic encephalopathy (Yes)	1.839	0.705	6.800	0.0091	1.579	25.08
HCC (No)	−2.447	1.616	2.294	0.130	0.004	2.054
Constant	−0.052	2.898	0.000	0.986		

CTP, Child–Pugh score; HCC, hepatocellular carcinoma; MELD, model for end‐stage liver disease; uNGAL, urinary neutrophil gelatinase‐associated lipocalin.

## Discussion

Results of this study showed that patients with HRS had intermediate uNGAL levels between the prerenal and intrinsic renal groups. Patients with or without infection, such as SBP, demonstrated no significant difference in uNGAL levels. However, patients with other complications of cirrhosis, such as hepatic encephalopathy, had higher uNGAL levels, and they were not statistically significant. In this study, uNGAL levels were significantly different in each group of AKI—the highest in the iAKI group, intermediate in the HRS group, low in the prerenal and CKD groups, and the lowest in patients with CLD without AKI. Values were higher in prerenal AKI than normal controls, but the difference was not significant (*P* −0.823). This will help to identify types of AKI on admission with single uNGAL values. sCr is poor in differentiating AKI types as low values were found for prerenal AKI, as well as for HRS and iAKI. Hence, the type of AKI could not be differentiated using creatinine alone.^4^ Even FeNa was higher in iAKI but<1 in both HRS and prerenal failure and hence was unable to differentiate prerenal from HRS. Hence, these data suggest that a single uNGAL level on admission will determine the type of AKI and help in its management. Nickolas et al.^25^ evaluated uNGAL levels in 635 unselected patients to assess whether a single measurement of uNGAL in a urine sample taken in the emergency room could detect AKI in a spectrum of patients. Patients with AKI had a median uNGAL of 416 l g/g. Creatinine was significantly higher in patients with AKI compared to values in patients with prerenal azotemia, CKD, or normal kidney function (30.1, 22.5, and 15.5 l g/g creatinine, respectively). In a longitudinal observational study, Verna et al.^22^ showed that uNGAL levels in HRS were intermediate between prerenal and iAKI. This was also confirmed by Claudia Fagundes et al.^21^

Our results showed higher uNGAL levels in patients with HRS and iAKI compared to previous studies. Renal tubular cells, especially distal nephron parts, synthesize and release NGAL in response to injury to the tubular epithelial cells in AKI. Hepatorenal syndrome is considered functional renal failure due to splanchnic vasodilatation and compensatory renal vasoconstriction; hence, NGAL values should be similar in the pre‐renal and HRS groups. However, most of the studies found higher NGAL values in the HRS group, suggesting more than just functional renal failure in HRS. In fact, pathologic investigations have shown subtle kidney tubular and glomerular damage in HRS kidneys, sometimes seen only on electron microscopy.^32^, ^33^ These are due to the cellular changes associated with chronic activation of angiotensin–aldosterone signaling. It is possible that profound renovascular constriction may cause subclinical tubular damage in at least a subset of nephrons, which is not detectable by urinary sodium and is not sensitive enough to detect mild or patchy tubular epithelial damage. These findings were supported by Cassinello Cet al,^34^ who reported that type 1 HRS could not be entirely functional in nature but may be associated with tubular damage. This may affect, to some extent, the response of this complication to treatment with terlipressin and albumin in patients with advanced cirrhosis. In addition, higher uNGAL levels in our study can be explained as follows: (i) uNGAL values were measured using the CMIA method, and values of uNGAL in AKI were not measured by this method in previous studies; hence, higher values can be due to the different techniques of measurement. One of the studies comparing uNGAL levels used two measurement methods enzyme linked immnunosorbent assay (ELISA) and CMIA, in healthy volunteers and showed higher values by CMIA methods, hence explaining the high values in our studies.^35^ (ii) Few studies published recently also had higher uNGAL levels that were comparable to our results where uNGAL levels were high in the iAKI group, 2625 ng/mL.^36^

In a small cross‐sectional study, Gerbes et al.^20^ evaluated the relationship between measured glomerular filtration rate (via 51Cr‐EDTA technique) with plasma and uNGAL in patients with decompensated cirrhosis and variable kidney function; this showed that a cut‐off of plasma NGAL>100 ng/mL was superior to creatinine in predicting patients with measured glomerular filtration rate (GFR) < 50 mL/min.

In our study, we showed that uNGAL levels >299 ng/mL and > 650 ng/mL had higher sensitivity and specificity than creatinine for identifying HRS from prerenal AKI and iAKI from HRS, respectively. Values between 299 and 650 ng/mL suggest HRS rather than prerenal or iAKI.

A study by Qaseem et al.^37^ used uNGAL cut‐off values of 286.3 𝜇g/g creatinine (area under ROC curve is 0.909) to differentiate HRS from ATN. Bassat et al.^38^ showed that uNGAL above 110 ng/mL had a sensitivity and specificity of 90% and 67%, respectively, for differentiating HRS from other types of AKI. In our study, higher uNGAL values were seen in patients with SBP (608 ng/mL *vs* 354.09 ng/mL, [*P* = 0.1031]) and encephalopathy (537 *vs* 343.5 ng/mL *P* = 0.07849), although they were not statistically significant compared to those without SBP or encephalopathy. Similar observations were made by Verna et al.^22^ This suggests that, although infections increase the chances of complications, uNGAL levels are not statistically different. However, contrasting results were reported by Claudia Fagundes,^21^ in which HRS with infection had higher uNGAL levels than patients without infection and was similar to those with ATN. Fagundes et al. included patients with a UTI, which we have excluded, and this may be the reason for increased values of uNGAL in their study as UTIs are known to increase uNGAL levels.

Literature review revealed some prognostic markers of mortality in patients with HRS. MELD was initially devised to predict survival in patients with complications of portal hypertension undergoing elective placement of transjugular intrahepatic portosystemic shunts. The principal use of the MELD score has been for the allocation of organs for liver transplantation. There are only few studies emphasizing the role of uNGAL in predicting mortality at 30 days and 90 days. In our study, patients who died at 30 days (short‐term transplant‐free mortality) had uNGAL values that were statistically different. Similar results have been reported by Barreto et al.^39^ On univariate analysis, uNGAL, MELD, length of stay, and sCr after 48 h were statistically significant between survivors *versus* nonsurvivors in the present study. Verna et al.,^22^ on univariate analysis, showed that uNGAL, sCr, and MELD predicted poor clinical outcome.

In the present study, on multivariate analysis, uNGAL and presence of hepatic encephalopathy were statistically significant in predicting mortality. On multivariate analysis, studies such as the one by Verna et al. have also showed that uNGAL >110 ng/mL and diagnosis of HRS were significant in predicting short‐term mortality. Gungor et al.^31^ showed that MELD‐Na and serum NGAL are independent predictors of mortality in a multivariate analysis. This could be because only patients with HRS were included. In our study, uNGAL levels correlated with mortality, and Gungor et al.^31^ showed serum NGAL correlated with mortality rather than uNGAL. In their study, plasma NGAL values >289.6 μg/dL had a shorter survival time compared with those with plasma NGAL levels <289.6 μg/dL.

### *Limitations*

The current study was performed in a single center, and this may be a limitation with respect to the potential generalization of our findings, which need to be validated and reproduced with a higher sample size. A larger study may also have allowed us to evaluate HRS types I and II individually. In addition, due to the small sample size, we could not correlate uNGAL levels in patients with HRS and infection such as SBP to those with acute tubular necrosis. Most of the diagnoses of iAKI were based on clinical and laboratory parameters, and kidney biopsy was performed only in two patients.

Another issue is that the current method for NGAL measurement, which uses a CMIA technique represents a logistical problem if results are to be used in clinical practice to take timely decisions. In this regard, a quick method for the measurement of NGAL (NGAL rapid) has been recently introduced, which will be valuable in clinical practice. Finally, the cost of the test is expected to decrease if it is regularly used in day‐to‐day practice.

In conclusion, our study shows that a single uNGAL measurement on admission helped to determine the type of AKI and prognostication in patients with cirrhosis with AKI. These findings, if confirmed in larger cohorts, could lead to the development of biomarker algorithms to rapidly identify patients with HRS and more accurately predict prognosis in such patients.

## Supporting information


**Figure S1.** Flow diagram for patient's enrollments and 30 day outcome.

**Table S1.** Post hoc Tukey multiple comparison test for analysis of variables in different groups of acute kidney injury
