# Supplementary figures and images for: Urinary neutrophil gelatinase‐associated lipocalin determines short‐term mortality and type of acute kidney injury in cirrhosis
Source: JGH Open. 2020 Jul 3;4(5):970–7. doi: 10.1002/jgh3.12377 (PMC7578274; doi:10.1002/jgh3.12377)

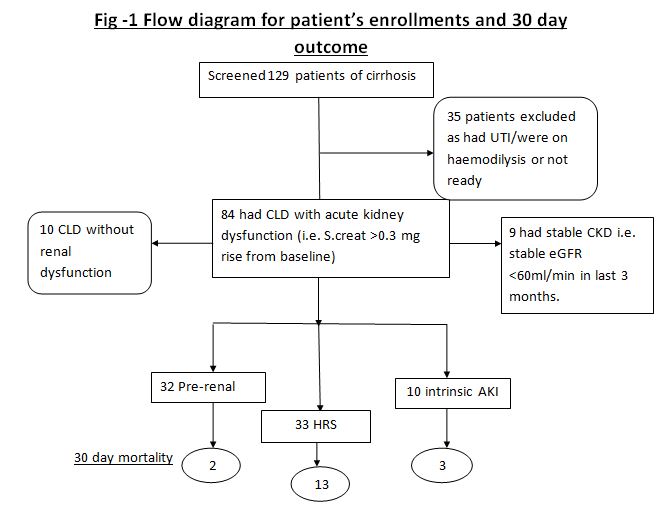

Supplement: Supplementary file 1 — Figure S1. Flow diagram for patient's enrollments and 30 day outcome. [file JGH3-4-970-s001.JPG]
