# Supplementary material for: Urinary neutrophil gelatinase‐associated lipocalin determines short‐term mortality and type of acute kidney injury in cirrhosis
Source: JGH Open. 2020 Jul 3;4(5):970–7. doi: 10.1002/jgh3.12377 (PMC7578274; doi:10.1002/jgh3.12377)
Supplement: Supplementary file 2 — Table S1. Post hoc Tukey multiple comparison test for analysis of variables in different groups of acute kidney injury [file JGH3-4-970-s002.docx]

| **Dependent Variable** | **Differential Diagnosis of AKI compared-** | **Difference** | **lower** | **Upper** | **p-value** | **Difference is-** |
| --- | --- | --- | --- | --- | --- | --- |
| **S.Creatinine** | **Intrinsic Renal vs. HRS** | 1.737 | 0.762 | 2.712 | 0.006 | Not Significant |
|  | **Intrinsic Renal vs. Prerenal** | 2.688 | 1.710 | 3.667 | 0.00001 | **Significant** |
|  | **HRS vs. Prerenal** | 0.951 | 0.280 | 1.621 | 0.00142 | **Significant** |
| **MELD** | **Intrinsic Renal vs. HRS** | 3.296 | -2.980 | 9.574 | 0.58933 | Not significant |
|  | **Intrinsic Renal vs. Prerenal** | 7.975 | 1.674 | 14.275 | 0.00591 | **Significant** |
|  | **HRS vs. Prerenal** | 4.678 | 0.363 | 8.992 | 0.02673 | **Significant** |
| **CTP** | **Intrinsic Renal vs. HRS** | 0.475 | -0.614 | 1.566 | 0.74292 | Not significant |
|  | **Intrinsic Renal vs. Prerenal** | 0.837 | -0.257 | 1.932 | 0.21651 | Not significant |
|  | **HRS vs. Prerenal** | 0.361 | -0.387 | 1.111 | 0.66481 | Not significant |
| **Urine NGAL** | **Intrinsic Renal vs. HRS** | 663.67 | 447.25 | 880.0 | 0.00001 | Not significant |
|  | **Intrinsic Renal vs. Prerenal** | 998.50 | 781.30 | 1215.0 | 0.00002 | **Significant** |
|  | **HRS vs. Prerenal** | 334.83 | 186.09 | 483.5 | 0.000003 | **Significant** |

**Supplementary table 1-Post hoc tukey multiple comparison test for analysis of variables in different groups of acute kidney injury**

[MELD-model for end stage liver disease,CTP-child pugh score,NGAL –neutrophil gelatinase associated lipocalin]
